# To vaccinate or not to vaccinate? The interplay between pro- and against- vaccination reasons

**DOI:** 10.1186/s12889-023-17112-6

**Published:** 2023-11-09

**Authors:** Marta Caserotti, Paolo Girardi, Roberta Sellaro, Enrico Rubaltelli, Alessandra Tasso, Lorella Lotto, Teresa Gavaruzzi

**Affiliations:** 1 Department of Developmental Psychology and Socialization, University of Padova, Padua, Italy; 2 Department of Environmental Sciences, Informatics and Statistics, Ca’ Foscari University of Venezia, Venice, Italy; 3 Department of Humanities, University of Ferrara, Ferrara, Italy; 4 Department of Medical and Surgical Sciences, University of Bologna, Bologna, Italy

**Keywords:** Pro-and against-reasons, Vaccination intention, Risk perception, Emotional competences

## Abstract

**Background:**

By mid 2023, European countries reached 75% of vaccine coverage for COVID-19 and although vaccination rates are quite high, many people are still hesitant. A plethora of studies have investigated factors associated with COVID-19 vaccine hesitancy, however, insufficient attention has been paid to the reasons why people get vaccinated against COVID-19. Our work aims to investigate the role of reasons in the decision to get vaccinated against COVID-19 in a representative sample of 1,689 adult Italians (March–April 2021) balanced in terms of age, gender, educational level and area of residence.

**Methods:**

Through an online questionnaire, we asked participants to freely report up to three reasons for and against COVID-19 vaccination, and the weight each had in the decision to get vaccinated. We first investigated the role of emotional competence and COVID-19 risk perception in the generation of both reasons using regression models. Next, we studied the role that the different reasons had in the vaccination decision, considering both the intention to vaccinate (using a beta regression model) and the decision made by the participants who already had the opportunity to get vaccinated (using a logistic regression model). Finally, two different classification tree analyses were carried out to characterize profiles with a low or high willingness to get vaccinated or with a low or high probability to accept/book the vaccine.

**Results:**

High emotional competence positively influences the generation of both reasons (ORs > 1.5), whereas high risk perception increases the generation of positive reasons (ORs > 1.4) while decreasing reasons against vaccination (OR = 0.64). As pro-reasons increase, vaccination acceptance increases, while the opposite happens as against-reasons increase (all *p* < 0.001). One strong reason in favor of vaccines is enough to unbalance the decision toward acceptance of vaccination, even when reasons against it are also present (*p* < 0.001). Protection and absence of distrust are the reasons that mostly drive willingness to be vaccinated and acceptance of an offered vaccine.

**Conclusions:**

Knowing the reasons that drive people’s decision about such an important choice can suggest new communication insights to reduce possible negative reactions toward vaccination and people's hesitancy. Results are discussed considering results of other national and international studies.

**Supplementary Information:**

The online version contains supplementary material available at 10.1186/s12889-023-17112-6.

## Introduction

By mid 2023 the European Union reached nearly 75% vaccine coverage for the primary vaccine cycle against COVID-19, with countries such as Croatia, Slovakia, and Poland falling short of 60% and others such as France, Portugal, and Italy close to 90% [1]. Although vaccination rates are, on average, quite high, many people are still hesitant. Vaccine hesitancy indicates the delay or refusal of a vaccine despite availability in vaccine services [2, 3] and is a multidimensional construct, resulting from the interaction between individual, social, and community aspects [4]. In the last two years, a plethora of studies have investigated factors associated with COVID-19 vaccine hesitancy showing, for example, that vaccine hesitancy is higher in women [5, 6], in young people [5, 7, 8], in people with low education [8, 9], low trust in authorities [10, 11], and strong conspiracy beliefs [5, 12, 13]. However, to the best of our knowledge no one has investigated the interplay that pro- and against- vaccination reasons may play in the choice to get vaccinated, namely what happens when a person has both pro- and against-vaccine considerations. Trying to fill this gap in the literature, our work aims to investigate how different reasons and the importance people place on them are likely to influence the decision to get vaccinated against COVID-19.

In line with the vaccine hesitancy *continuum* defined by SAGE [2], while extremely pro-vax people are likely to express only reasons pro-vaccination and extremely no-vax people are likely to express only reasons against vaccination, individuals who fall between the two extreme end-points are likely to feel some doubts. This large number of people offer us the unique opportunity to assess which category of reasons (pro- vs. against- vaccination) is more impactful in driving people's vaccination decisions. As it is reasonable to imagine, among the reasons for choosing to get (or not) vaccinated some reasons are more rational, while others are more related to affect. For example, there are people who rationally recognize the importance of vaccines but at the same time are frightened by the side effects. Thus, the decision to get (or not) vaccinated is the result of a complex process, in which costs and benefits are weighed more or less rationally. Indeed, while several studies have pointed out that the decision to vaccinate is due to cognitive rather than emotional processes [14–17], others have highlighted the role of affect and risk perception in the vaccination decision [18–20]. Thus, the intention to accept the vaccine is driven by emotional and affective feelings as much as by cognitive and rational judgments. Particular attention to what people feel and think about vaccine-preventable diseases and vaccination in general is paid in the model developed by the “Measuring Behavioral and Social Drivers of Vaccination” (BeSD), a global group of experts established by the World Health Organization [21]. This model encompasses two groups of proximal antecedents of vaccination, namely, what people think and feel (e.g., perceived risk, worry, confidence, trust and safety concerns) and social processes (e.g., provider recommendation, social norms and rumors). Antecedents affect vaccination motivation (i.e., vaccination readiness, willingness, intention, hesitancy), which can then be strengthened or weakened by practical issues (such as vaccine availability, convenience and cost but also requirements and incentives), resulting in acceptance, delay or refusal of vaccination (vaccination behavior).

Although some studies have considered whether the cognitive or affective component has greater weight in determining the intention to vaccinate, no one, to the best of our knowledge, has studied the interplay between pro- and against- vaccination reasons, nor the weight these have in the choice to vaccinate. In addition to the drivers already studied in the literature [5–8, 11, 12], we believe that the focus on this interaction may be relevant to better understand the complex phenomena related to vaccine hesitancy. Few recent studies have attempted to investigate the complexity of vaccination choice by studying the reasons why people choose to get (or not) vaccinated against COVID-19. Fieselmann and colleagues [22] highlighted that among the reasons that reduce adherence to vaccination are a low perception of its benefits, a low perception of the risk of contracting COVID-19, health concerns, lack of information, distrust of the system, and spiritual or religious reasons. Another study, instead, shed light on the reasons that encourage hesitant people to consider vaccination, such as protecting themselves, their family, friends and community from COVID-19, and being able to return to normal life [23].

In the present study we asked the participants to spontaneously come up with their own reasons to get (or not) vaccinated, without limiting or influencing them with a set of predefined options to choose from, thus aiming to obtain more genuine answers that may better capture the intuitive aspect of people’s opinions (for a similar reasoning see [24]). The procedure we used has been implemented by Moore et al. [23], the only study, as far as we know, that asked for reasons with an open-ended question. Critically, in their study, participants were asked to report only reasons in favor of vaccination (e.g., "What are your reasons for getting the COVID-19 vaccine?"), excluding reasons against. By contrast, we asked participants to freely report up to three reasons in favor and up to three reasons against COVID-19 vaccination and to rate on a 5-point Likert scale their weight in the decision about getting (or not) vaccinated.

From a theoretical point of view, the reasons pro- and against vaccination may be seen within the framework of prospect theory [25, 26] which suggests that people evaluate the outcome of a choice based on a reference point, against which losses and gains are determined: the former below this point, the latter above this point. Importantly, especially in this specific context, losses and negative consequences are weighted more than gains and benefits, making us hypothesize that if a person has one reason for and one reason against the vaccine, which are of equal importance, they will more likely lean toward choosing not to vaccinate. Consistently, it is known that negative experiences have a greater impact than neutral or positive ones (i.e., the negativity bias [27]).

Besides delving into the reasons that may influence the choice to get (or not) vaccinated, it would be interesting to also look at the individual differences that may determine the reporting of pro- and against- vaccination reasons and their valence. In this regard, the literature suggests that risk perception and emotion regulation can both have a great impact in the decision to get vaccinated. For instance, studies conducted during H1N1 influenza have shown that perception of disease-related risk is one of the strongest predictors of vaccine adherence [28, 29]. Additional insights have been provided by more recent studies investigating the role of COVID-19 risk perception in the decision to get vaccinated against COVID-19. Viswanath and colleagues [30] showed that people are more willing to vaccinate themselves and those under their care to the extent to which they feel more vulnerable to COVID-19 and rate the consequences of a possible infection as severe. Such a relationship between COVID-19 risk perception and intention to vaccinate was confirmed by another study using a cross-sectional design, which focused on the early months of the pandemic [31]. This study also examined how risk perception changed during the pandemic phases and showed that during the lockdown, compared to the pre-lockdown phase, also those who reported some hesitancy were more likely to get vaccinated when they perceived a strong COVID-19 risk.

With regard to emotion regulation, the literature suggests that people react differently to affective stimuli [32] and that their decisions are influenced by their abilities to regulate emotions [33, 34]. Recent works investigating the relationship between hesitancy in pediatric vaccinations and the emotional load associated with vaccinations, have shown that a negative affective reaction is one of the factors leading to lower vaccine uptake [35, 36]. Specifically, Gavaruzzi and colleagues [36] showed that concerns about vaccine safety and extreme views against vaccines are associated with vaccine refusal. Interestingly, they also showed that parents' intrapersonal emotional competences, i.e., their ability to manage, identify, and recognize their own emotions, is critical to vaccine acceptance for their children. Therefore, in our study we measured people's risk perception and emotional competencies to assess their possible role in the production of reasons in favor and against vaccination.

**Fig. 1:**
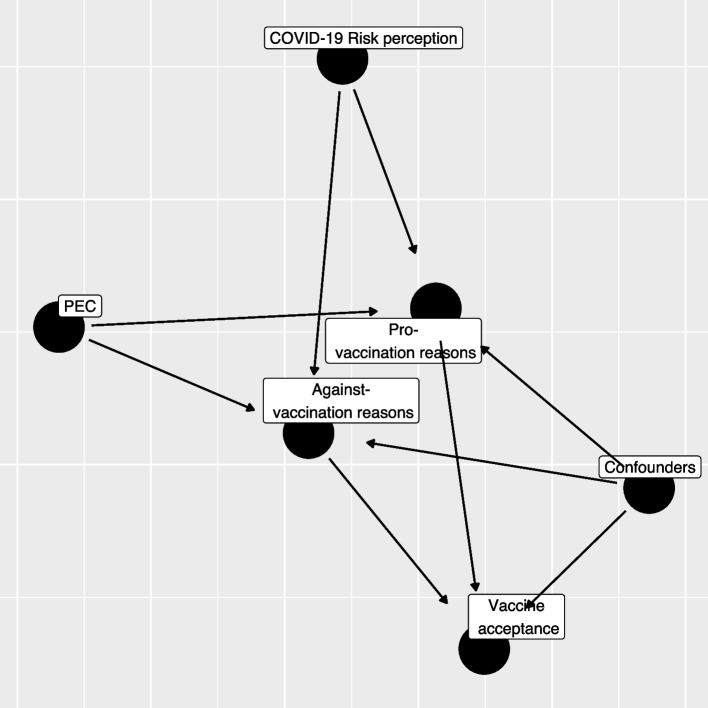
Directed Acyclic Graph (DAG) between variables considered in the study (PEC: Short Profile of Emotional Competence scale)

As described in Fig. 1, the relationship between different domains of interest can be hierarchically structured, using a directed acyclic graph, starting from the risk perception and emotion regulation, to the generation of pro- and against- vaccination reasons and their valence, and finally to the vaccination willingness/adherence. With respect to the mentioned structure, we are interested to investigate the following research hypotheses:

1. The number and weight associated with reasons pro- and against-vaccination should be influenced by individual differences in the ability to regulate emotions;
2. The number and weight associated with pro-vaccination reasons should be influenced by individual differences in COVID-19 risk perception;
3. A higher number of strong (i.e., with high weight) reasons pro- (vs. against-) vaccination should correspond to a more (vs. less) likelihood to accept the vaccination.
4. Generating an equal number of reasons in favor and against vaccination should lead to a weaker likelihood to accept the vaccination.

As we conducted the study between March and April 2021, a time when vaccinations were being progressively rolled out, we decided to consider the role of personal reasons on both the intention to get vaccinated (for those who had not yet had the opportunity to get vaccinated) and the choice already made (e.g., vaccine received or booked vs. refused).

Finally, through a non-parametric classification analysis, we will explore how specific pro- and against-vaccination reasons impact the decision to get (or not) vaccinated. Specifically, we will investigate the role that different categories of reasons play in the choice to vaccinate.

## Methods

### Participants

Data collection was commissioned to a survey and market research agency (Demetra Opinions.net), with the aim of securing a representative sample of the adult (+ 18) Italian population, estimated at 49.8 million [37]. The sample was balanced in terms of age, gender, educational level (middle school or lower, high school, degree or higher), and area of residence (North, Center, South, and Islands). The agency distributed via email the survey link to its panelists, who freely decided whether to participate in the study in exchange for financial compensation. Out of 1,833 participants who started the questionnaire, 77 (4%) were excluded because they did not complete the survey and 16 (0.9%) were excluded since they reported offensive content in open-ended questions. Finally, 124 (6.8%) participants were excluded because of missing information. Thus, the final sample consisted of 1,689 participants. The project was approved by the ethical committee for Psychology Research of the University of Padova (Italy), with protocol number 3911/2020 and informed consent was obtained for all participants.

### Procedure

We developed an *ad-hoc* questionnaire including a series of open-ended and closed questions (see Additional file 1: Appendix 2 for the full material). We first investigated the vaccination status of the participants, asking whether they already had received at least the first dose, whether they had booked it or were still ineligible, and finally whether they had refused the vaccination. Those not yet eligible were asked to rate how likely they would be to get vaccinated at the time they responded (0 = *Not at all likely*, 100 = *Extremely likely*). Then, we asked participants to report a maximum of three reasons both in favor of the COVID-19 vaccine and against it (in counterbalanced order) and to rate how much each of the reported reasons weighed in their choice to vaccinate or not, on a 5-point likert scale (1 = *Not at all*, 5 = *Extremely*). Due to the sparsity on the rate and the number of provided reasons we re-coded the provided information into two semi-quantitative variables, one for pro- and one for against- vaccination reasons, as following: missing/invalid reasons, low average rating (answers 1–3 on the Likert scale) and 1–3 reasons, high rating (answers 4–5 points on the Likert scale) and 1 reason, and high average rating (answer 4–5 points on the Likert scale) and 2–3 reasons.

The questionnaire also included the 20-item Short Profile of Emotional Competence scale (S-PEC; [38]) to measure intra- and inter-personal emotional competences separately. The intra-personal scale (10 items) refers to emotional competences related to oneself and it includes items such as "In my life I never make decisions based on my emotions'' or "I don't always understand why I react in a certain way". The inter-personal scale (10 items) refers to emotional competences related to other people and it includes items such as “If I wanted, I could easily make someone feel uneasy” or “Most of the time, I understand why the people feel the way they do”. All items are answered on a 7-point likert scale (1 = *Not at all agree*, 7 = *Completely agree*). The internal consistency of the S-PEC scale, measured by means of Cronbach’s α, was adequate (α = 0.81). Further, we measured participants' risk perception of COVID-19 by asking them to indicate how scared they felt of the virus, how serious they think the disease is, how likely they think they are to get sick, and how worried they feel about the various mutations [10, 31]. We then asked participants to report their age, gender, educational level, their occupation (health workers, white-collar workers, entrepreneurs, other non-health-related contract forms, and the unemployed), whether they already had COVID-19 (No or don't know, Yes asymptomatic, Yes with few symptoms, and Yes with severe symptoms). The questionnaire was pilot tested by 30 participants who filled the questionnaire first then were asked to discuss and comment on the comprehension of the wording of questions and answer options. Two questions were slightly reworded to improve clarity.

### Scoring of reasons

In the first instance, a bottom-up process from reasons to categories was followed by reading a sample of both types of reasons, with the aim of constructing initial categorizing patterns. Examples of pro-vaccination reasons include protection of personal and public health, return to normality, and civic duty; while reasons against vaccination include fears for one's health, sociopolitical perplexity, and distrust of science and institutions (see Additional file 1: Appendix 1). At this stage, response information was added to the categorizations indicating whether the responses were valid or missing/invalid. Specifically, valid responses had both a reason and the respective weight; missing/invalid responses were those where reason, weight or both were missing or with utterly unrelated concepts or meaningless strings or letters. Finally, by applying a top-down process, we constructed macro categories by merging specific conceptually assimilated categories, so as to avoid the dispersion of data into too many ramifications (see Table S5).

### Statistical analysis

#### Descriptive analysis

All the analyses were performed only on respondents with no missing observations on the variables of interest (1,681, 92%) excluding also a limited number of those with a non-valid set of pro- or against-vaccination reasons (Table S1; 0.9%). The study variables were summarized in frequency tables and figures (frequency for categorical variables, median and Interquartile Range (IQR) for continuous variables). Kruskal–Wallis tests were computed to compare the distribution of continuous variables across the categories of vaccine status. Categorical variables were compared using chi-squared or Fisher's exact test where expected frequencies in any combination were less than 10. Statistical significance was assumed at the 5% level.

#### COVID-19 Perceived risk—exploratory factor analysis

An Exploratory Factorial Analysis (EFA) was performed on groups of variables related to COVID-19 perceived risk: scare, severity, contagiousness, and the likelihood of mutation. Since the presence of limited support (0–100 scale) and non-normal marginal distribution, the EFA was performed using a weighted least square mean and variance adjusted (WLSMV) estimator. We extracted from the EFA only the first factor, which explained the highest percentage of variance (Table S2; 61%). The estimated loadings were then used to calculate the regression factor scores. The number and the name of items included, their internal consistency (Cronbach’s α), the estimated loadings, and the proportion of deviance explained are reported in Table S2.

#### Propensity score weighting

At the time of data collection (March–April 2021), the vaccine offer was not opened to the entire population. To adjust the estimates of the following regression models for the propensity to receive the vaccine, we estimated a logistic regression model in which the dependent variable was the response to the question about a previous vaccination offer (Yes/No), while all the factors that can influence the vaccine proposal served as independent variables: age-class (young ≤ 25, young adult 26–45, adult 46–65, elderly 66–84), gender (male, female), occupational status (health worker, not at work, not health worker-employer, not health worker-entrepreneur, not health worker-other), educational level (low = middle school or lower, medium = high school, high = degree or higher), key worker status (yes, no, I don’t know), past COVID-19 contagion (no, yes asymptomatic, yes low symptoms, yes severe symptoms), and familiar status (single/in a relation, married/cohabitant, divorced/separated/other). The predicted probability was used to estimate the weights for the following regression models using a framework based on an inverse probability of treatment weighting (IPTW; for further details, see [39]).

#### Regression models

Our research questions can be summarized by trying to describe the relationship exploited by the directed acyclic graph in Fig. 1. The first step regression model aims to assess how S-PEC scores (inter- and intra-personal) and COVID-19 risk perception influenced the reasons pro- and against-vaccination produced by participants while considering the presence of a set of confounders (age-class, gender, occupational status, educational level, key worker status, and familial status).

Since both the pro- and against-vaccination reasons are formed by a categorical variable with 4 levels (missing/invalid, low 1/2/3 reasons, high 1 reason, high 2/3 reasons), we evaluated whether S-PEC and COVID-19 risk perception scores influenced the distribution of pro- and against-vaccination reasons employing two different multinomial regression models including all the previously mentioned variables (S-PEC, COVID-19 risk perception, and confounders). The overall significance of a variable in the model was tested using an analysis of the variance (ANOVA).

The second step in the analyses was taken to investigate whether the generation of pro- and/or against-vaccination reasons affected the willingness to be vaccinated or the vaccine acceptance. Each participant reported their willingness to get vaccinated on a 0–100 scale or, in case a COVID-19 vaccine had been already offered, their vaccination status (done, booked, or refused). For respondents who had not yet been contacted for booking/getting the vaccination, we evaluated whether pro- and/or against vaccination reasons influenced the willingness to be vaccinated by employing a beta regression model in which the respondent variable scale (0–100) was rescaled to be a relative frequency [40]. The full models included the semi-quantitative pro- and against-vaccination reasons variables and, even if non-statistically significant, all the confounders in order to adjust for age class, gender, educational level, occupational status, familial status, and key worker status. Beta regression coefficients were estimated using a maximum likelihood estimator (MLE). Results were presented in terms of Odds Ratios (ORs) by exponentiating the estimated coefficients and producing a relative 95% Confidence Interval (95% CI).

A further regression analysis was conducted through a logistic regression model to explain which factors influenced vaccine acceptance (done/booked vs. refused) among those who already received the vaccine offers. The full model included the same variables considered in the previous beta regression model, after recoding the variables related to pro- and against-vaccination reasons into a binary form (missing/invalid vs. presence of at least one valid reason) due to low sample size and the sparsity of the response variable. As a consequence, we tested a simplified version of Hypothesis 3, considering the presence (vs. missing/invalid) of pro- or against-vaccination reasons in order to test their influence on the probability of having accepted/booked the vaccination.

Results were reported employing ORs and relative 95% Confidence Interval (95% CI).

Both the beta regression and logistic regression were weighed using an IPTW scheme to take into account the presence of a different probability of a vaccine offer among respondents.

The presence of an interaction between pro- and against-vaccination reasons was tested by means of a likelihood ratio test. The regression models were estimated through the R 4.0 program (R Core Team, 2021), and for the beta regression we employed the *betareg* package [41].

#### Classification tree analysis

Two different classification tree analyses were carried out to characterize profiles with a low or high willingness to get vaccinated (respondents who had not yet been offered a vaccine) or with a low or high probability to accept/book the vaccine (respondents who had already received a vaccine offer).

Although the dependent variables were non-normally distributed (scale 0–100 or binary 0/1), we considered them continuously distributed adopting a splitting criterion based on the analysis of the variance (ANOVA). We tested the inclusion in the model considering the type of pro- or against-vaccination reasons. A tree pruning strategy was adopted to reduce classification tree overfitting considering the overall determination coefficient (R^2^) as an indicator and fixing that at each classification step in the tree if the R^2^ did not increase by 0.5% the tree should be stopped. Classification tree analysis was performed using the *rpart* package [42] on R environment [43].

## Results

### Descriptive analysis

The main characteristics of the respondents by vaccination status (received, booked, not yet, and refused) were reported in Table 1. Among respondents, 23.3% were offered the vaccination and, among them, 13.8% refused it (Fig. S1). Among those not yet eligible, willingness to be vaccinated showed a median value of 80 points (average: 68.7). The distribution of gender was almost equal (51% females, 49% male), and the median age was 47 years old (IQR: 34–57 years). Educational level was low in 41% of the sample, while the most represented employment status was not at work (39%) followed by employed (37%), and entrepreneur (9.8%). A quarter (26%) of respondents classified themselves as key workers during the COVID-19 pandemic. The predominance of respondents (63%) were married or living with a partner, while only 9% had had a COVID-19 infection.

**Table 1:** Main characteristics of the participants, overall and by COVID-19 vaccine status

**Characteristics**	**Overall**, *N* = 1,681^*1*^	**COVID-19 vaccine status**				***P*-value**^*2*^
		**Booked**, *N* = 159^*1*^	**Done**, *N* = 179^*1*^	**Not yet**, *N* = 1,289^*1*^	**Refused**, *N* = 54^*1*^	
**Gender**						0.222
Male	851 (51%)	88 (55%)	100 (56%)	636 (49%)	27 (50%)	
Female	830 (49%)	71 (45%)	79 (44%)	653 (51%)	27 (50%)	
**Age-class (years)**						< 0.001
≤ 25	196 (12%)	12 (7.5%)	13 (7.3%)	160 (12%)	11 (20%)	
26–45	605 (36%)	45 (28%)	51 (28%)	484 (38%)	25 (46%)	
46–65	705 (42%)	64 (40%)	83 (46%)	543 (42%)	15 (28%)	
66–84	175 (10%)	38 (24%)	32 (18%)	102 (7.9%)	3 (5.6%)	
**Educational level**						< 0.001
Low	684 (41%)	50 (31%)	47 (26%)	561 (44%)	26 (48%)	
Middle	666 (40%)	77 (48%)	63 (35%)	506 (39%)	20 (37%)	
High	331 (20%)	32 (20%)	69 (39%)	222 (17%)	8 (15%)	
**Occupational status**						< 0.001
Health worker	94 (5.6%)	16 (10%)	50 (28%)	26 (2.0%)	2 (3.7%)	
Not at work	663 (39%)	67 (42%)	44 (25%)	529 (41%)	23 (43%)	
Not health worker – Employer	616 (37%)	49 (31%)	72 (40%)	475 (37%)	20 (37%)	
Not health worker – Entrepreneur	165 (9.8%)	14 (8.8%)	7 (3.9%)	139 (11%)	5 (9.3%)	
Not health worker – Other	143 (8.5%)	13 (8.2%)	6 (3.4%)	120 (9.3%)	4 (7.4%)	
**Key worker status**						0.007
Yes	445 (26%)	27 (17%)	39 (22%)	361 (28%)	18 (33%)	
No	1,074 (64%)	110 (69%)	119 (66%)	817 (63%)	28 (52%)	
I don’t know	162 (9.6%)	22 (14%)	21 (12%)	111 (8.6%)	8 (15%)	
**Familial status**						0.006
Single/In a relation	421 (25%)	45 (28%)	92 (51%)	268 (21%)	16 (30%)	
Married/Cohabitant	1,059 (63%)	103 (65%)	69 (39%)	857 (66%)	30 (56%)	
Divorced/Separated/Other	201 (12%)	11 (6.9%)	18 (10%)	164 (13%)	8 (15%)	
**Past COVID-19 contagion**						0.088
No, I don’t know	1,532 (91%)	140 (88%)	162 (91%)	1,177 (91%)	53 (98%)	
Yes, asymptomatic	37 (2.2%)	5 (3.1%)	9 (5.0%)	23 (1.8%)	0 (0%)	
Yes, low symptoms	104 (6.2%)	12 (7.5%)	8 (4.5%)	83 (6.4%)	1 (1.9%)	
Yes, severe symptoms	8 (0.5%)	2 (1.3%)	0 (0%)	6 (0.5%)	0 (0%)	
**COVID-19 perceived risk**						< 0.001
Low	552 (33%)	47 (30%)	47 (26%)	423 (33%)	35 (65%)	
Medium	561 (33%)	59 (37%)	72 (40%)	421 (33%)	9 (17%)	
High	568 (34%)	53 (33%)	60 (34%)	445 (35%)	10 (19%)	
**S-PEC self**						0.184
Low	657 (39%)	64 (40%)	78 (44%)	491 (38%)	24 (44%)	
Medium	521 (31%)	58 (36%)	47 (26%)	398 (31%)	18 (33%)	
High	503 (30%)	37 (23%)	54 (30%)	400 (31%)	12 (22%)	
**S-PEC others**						0.078
Low	594 (35%)	47 (30%)	69 (39%)	455 (35%)	23 (43%)	
Medium	522 (31%)	58 (36%)	40 (22%)	410 (32%)	14 (26%)	
High	565 (34%)	54 (34%)	70 (39%)	424 (33%)	17 (31%)	

^1^Median (IQR) or Frequency (%)

^2^Fisher’s Exact Test for Count Data with simulated *p*-value (based on 2000 replicates)

COVID-19 risk perception and the S-PEC score (intra- and inter-personal) were categorized into three categories according to empirical tertiles (low:1^st^ tertile, medium: 2^nd^ tertile, high: 3^rd^ tertile). The level of COVID-19 risk perception differed across vaccination status (*p* < 0.001). The reasons pro- and against-vaccination have a different distribution according to COVID-19 vaccination status (Table 2). The highest frequency of pro-vaccination reasons was reported by those who received the COVID-19 vaccination; conversely the lowest frequency of pro-vaccination reasons was generated by those who refused the vaccine, whereas, intermediate frequencies were shown by people who were not yet offered the vaccination and those who had booked the vaccine, who reported a comparable distribution of the number of pro-vaccination reasons. A reverse pattern was exhibited for against-vaccination reasons, which were generated with the highest percentage by respondents who refused the vaccine (in particular high and multiple reasons). Conversely those who have booked/done the COVID-19 vaccine showed the lowest frequency of reasons against vaccination, while respondents without a vaccine offer reported an intermediate frequency of reasons against vaccination.

**Table 2:** Number and strength of pro and against-vaccination reasons, overall and by COVID-19 vaccine status

**Reasons**	**Overall**, *N* = 1,681^*1*^	**COVID-19 vaccine status**				***P*-value**^*2*^
		**Booked**, *N* = 159^*1*^	**Done**, *N* = 179^*1*^	**Not yet**, *N* = 1,289^*1*^	**Refused**, *N* = 54^*1*^	
**Pro-vaccination**						< 0.001
Missing—Invalid	581 (35%)	63 (40%)	40 (22%)	440 (34%)	38 (70%)	
Low; 1/2/3 reasons	73 (4.3%)	3 (1.9%)	6 (3.4%)	61 (4.7%)	3 (5.6%)	
High; 1 reason	608 (36%)	59 (37%)	82 (46%)	460 (36%)	7 (13%)	
High; 2/3 reasons	419 (25%)	34 (21%)	51 (28%)	328 (25%)	6 (11%)	
**Against-vaccination**						< 0.001
Missing—Invalid	748 (44%)	86 (54%)	96 (54%)	541 (42%)	25 (46%)	
Low; 1/2/3 reasons	334 (20%)	36 (23%)	53 (30%)	236 (18%)	9 (17%)	
High; 1 reason	397 (24%)	29 (18%)	23 (13%)	335 (26%)	10 (19%)	
High; 2/3 reasons	202 (12%)	8 (5.0%)	7 (3.9%)	177 (14%)	10 (19%)	

^1^Median (IQR) or Frequency (%)

^2^Pearson's Chi-squared test

### Propensity score weighting

The estimated results of the propensity score model for the vaccine offer are shown in Table S3. Respondents older than 65 years exhibited a nearly four-fold increase in the probability to be contacted for the vaccination with respect to the reference age-class (≤ 25 years). All non-health employees showed a high drop in the probability of having received the vaccination offer, while the probability increased as the educational level increased. Being a key worker during pandemic resulted in an increased probability of having received the vaccination proposal while no statistical significant influence was observed for the past COVID-19 contagion or for familial status. The distribution of the propensity score by vaccine status obtained by the model is reported in Fig. S1, in which it is shown that the distribution is different by vaccine offer, but the two density functions partially overlap. The discriminant power of the propensity score estimated was only discrete (ROC analysis, AUC: 71.8%).

### Regression models

The results of the multinomial regression models which investigated the effect of emotional competences and risk perception on the generation and the predictors of pro- and against-vaccination reasons with respect to missing/invalid level and the reference categories are presented in Table 3 (see also Fig. 1). Compared to the reference category (missing/invalid), high values of S-PEC-self were associated with a higher probability to report pro- and against-vaccination reasons (all ORs > 1.5), while high values of S-PEC-others were associated with a mild probability to report multiple pro-vaccination reasons (all ORs > 1.42). A high (vs. low) COVID-19 risk perception increased the frequency of one strong pro-vaccination reason while it had a null or low decremental effect on the frequency of against weak vaccination reasons. Further, medium (vs. low) COVID-19 risk perception only increased the strong pro-vaccination. Compared to the reference age-class (young), adults and elderly showed a higher probability to generate a strong unique pro-vaccination reason (adults vs. young OR: 1.72, 95%CI: 1.07–2.77); elderly vs. young OR: 2.24, 95%CI: 1.26–4.00), while lower probability to generate against vaccination reasons was observed for elderly compared to young respondents (OR: 0.48, 95%CI: 0.26–0.90). Female participants generated fewer strong pro-vaccination reasons (ORs < 0.73), and also fewer multiple weak against-vaccination reasons (OR: 0.68, 95%CI: 0.51–0.91) compared to male participants. Overall, the occupational status did not affect the generation of pro- and against-vaccination reasons (ANOVA test *p* > 0.05); however an increased frequency of low 1/2/3 against-vaccination reasons emerged among the category “Other—not health workers” compared to the reference group represented by health workers (OR: 2.52, 95%CI:1.09–5.86). Pro-vaccination reasons are more frequent as the educational level becomes higher, while the relation of the educational level with against- vaccination reasons appears weaker and significantly increased only for the presence of multiple weak reasons against vaccination (High vs. Low educational level, OR: 2.10, 95%CI: 1.45–3.03). Not being a key worker is related to a higher frequency of multiple strong both pro- and against vaccination reasons. The familiar status did not seem to be related to the frequency or the strength of the reasons, except for the status of divorced/separate/other that, with respect to the reference category single/in a relation, showed a twofold increase in the frequency of a strong unique against vaccination reason.

**Table 3:** Odds ratios (ORs) and relative 95%CI (Confidence Interval) estimated by two distinct multinomial models for pro- and against-vaccination reasons respect to missing/invalid level and the reference categories^a^. Statistically significant results are represented in bold

	***Pro-vaccination reasons***			***Against-vaccination reasons***		
*Strength* *Number of motivations*	*Low* *1/2/3 Motiv*	*High* *1 Motiv*	*High* *2/3 Motiv*	*Low* *1/2/3 Motiv*	*High* *1 Motiv*	*High* *2/3 Motiv*
**Characteristics**	**OR (95%CI)**	**OR (95%CI)**	**OR (95%CI)**	**OR (95%CI)**	**OR (95%CI)**	**OR (95%CI)**
S-PEC self [Medium]	0.94 (0.50 – 1.75)	1.01 (0.76 – 1.34)	1.09 (0.79 – 1.51)	0.88 (0.63 – 1.22)	1.17 (0.86 – 1.59)	0.89 (0.60 – 1.33)
S-PEC self [High]	1.81 (0.94 – 3.48)	**1.51 (1.10 – 2.09)**	**2.02 (1.42 – 2.88)**	**1.46 (1.02 – 2.09)**	**1.76 (1.25 – 2.46)**	**1.62 (1.07 – 2.47)**
S-PEC others [Medium]	0.87 (0.47 – 1.61)	1.10 (0.82 – 1.46)	1.26 (0.91 – 1.75)	0.90 (0.65 – 1.26)	0.89 (0.65 – 1.22)	0.90 (0.60 – 1.35)
S-PEC others [High]	0.83 (0.43 – 1.61)	1.29 (0.95 – 1.77)	**1.42 (1.00 – 2.01)**	1.19 (0.84 – 1.69)	1.08 (0.78 – 1.50)	1.27 (0.84 – 1.92)
COVID-19 risk perception [Medium]	1.11 (0.62 – 1.99)	**1.43 (1.06 – 1.91)**	**1.45 (1.05 – 1.99)**	1.25 (0.91 – 1.72)	1.05 (0.76 – 1.43)	0.92 (0.62 – 1.35)
COVID-19 risk perception [High]	0.77 (0.41 – 1.47)	**1.55 (1.15 – 2.08)**	1.38 (0.99 – 1.91)	**0.64 (0.45 – 0.91)**	0.99 (0.72 – 1.35)	0.76 (0.51 – 1.12)
Age-class [26-45]	0.67 (0.28 – 1.61)	1.18 (0.74 – 1.86)	**0.60 (0.38 – 0.96)**	0.66 (0.41 – 1.08)	0.79 (0.49 – 1.26)	0.69 (0.39 – 1.20)
Age-class [46-65]	1.24 (0.51 – 3.03)	**1.72 (1.07 – 2.77)**	0.72 (0.44 – 1.16)	1.01 (0.61 – 1.67)	0.82 (0.50 – 1.34)	0.68 (0.38 – 1.21)
Age-class [66–84]	1.44 (0.48 – 4.30)	**2.24 (1.26 – 4.00)**	0.87 (0.47 – 1.59)	1.24 (0.69 – 2.23)	**0.48 (0.26 – 0.90)**	0.49 (0.23 – 1.04)
Gender [Female]	1.02 (0.60 – 1.75)	**0.73 (0.56 – 0.94)**	**0.69 (0.52 – 0.92)**	**0.68 (0.51 – 0.91)**	0.99 (0.76 – 1.30)	0.98 (0.69 – 1.38)
Occupational Status [Not at work]	0.73 (0.22 – 2.38)	1.16 (0.62 – 2.18)	1.12 (0.56 – 2.22)	2.00 (0.95 – 4.19)	1.58 (0.80 – 3.12)	1.30 (0.52 – 3.25)
Occupational Status [Not health worker – employer]	0.52 (0.17 – 1.55)	1.29 (0.73 – 2.30)	1.11 (0.59 – 2.10)	1.79 (0.90 – 3.56)	1.69 (0.90 – 3.17)	1.40 (0.59 – 3.30)
Occupational Status [Not health worker – Entrepreneur]	0.45 (0.12 – 1.71)	1.01 (0.52 – 1.95)	0.81 (0.39 – 1.69)	1.77 (0.82 – 3.84)	1.45 (0.70 – 2.97)	1.03 (0.38 – 2.79)
Occupational Status [Not health worker – Other]	0.63 (0.15 – 2.56)	1.45 (0.71 – 2.96)	1.29 (0.58 – 2.86)	**2.52 (1.09 – 5.86)**	1.32 (0.60 – 2.92)	2.18 (0.80 – 5.93)
Educational level [Medium]	1.26 (0.72 – 2.21)	**1.69 (1.29 – 2.20)**	**1.58 (1.17 – 2.14)**	1.34 (0.98 – 1.83)	**1.41 (1.06 – 1.86)**	1.08 (0.75 – 1.55)
Educational level [High]	1.46 (0.70 – 3.02)	**2.14 (1.51 – 3.04)**	**2.96 (2.03 – 4.29)**	**2.10 (1.45 – 3.03)**	1.28 (0.88 – 1.85)	1.29 (0.82 – 2.03)
Key worker status [No]	1.31 (0.65 – 2.66)	1.24 (0.91 – 1.70)	**1.49 (1.04 – 2.13)**	1.12 (0.78 – 1.61)	1.32 (0.94 – 1.84)	**1.65 (1.04 – 2.61)**
Key worker status [I don't know]	0.34 (0.09 – 1.27)	1.17 (0.76 – 1.79)	0.95 (0.57 – 1.59)	1.00 (0.60 – 1.65)	1.00 (0.62 – 1.62)	1.47 (0.80 – 2.70)
Familial status [Married/Cohabitant]	0.72 (0.38 – 1.36)	0.92 (0.67 – 1.25)	1.01 (0.72 – 1.44)	0.85 (0.60 – 1.20)	1.38 (0.98 – 1.95)	0.92 (0.61 – 1.40)
Familial status [Divorced/Separate/Other]	0.56 (0.20 – 1.53)	0.86 (0.53 – 1.39)	0.82 (0.47 – 1.42)	0.90 (0.51 – 1.57)	**1.90 (1.14 – 3.17)**	1.44 (0.77 – 2.70)

^a^reference category: S-PEC self [Low], S-PEC others [Low], C19 risk perception [Low], age-class [≤ 25], Gender [Male], Occupational status [Health worker], Educational level [Low], Key worker [Yes], Familial status [Single/In a relation]

**Fig. 2:**
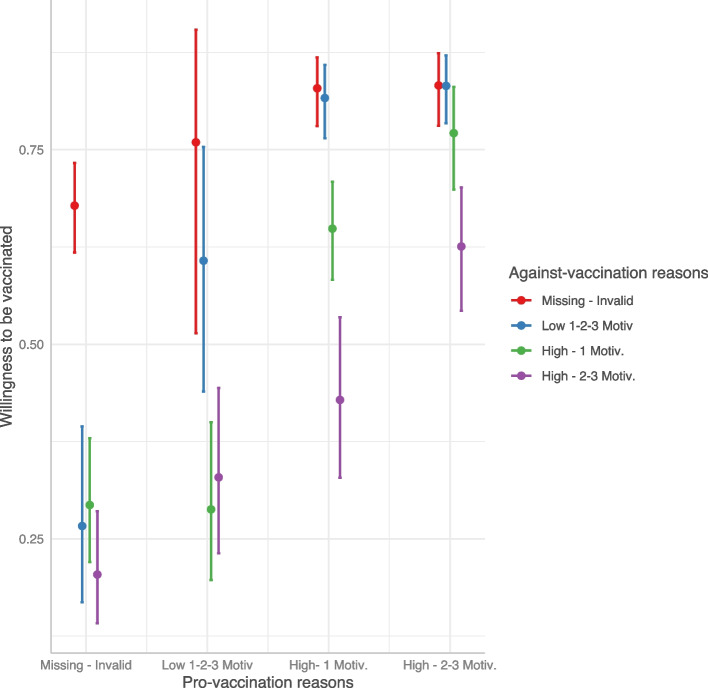
Predicted willingness to get vaccinated by interaction between pro- and against-vaccination reasons

Through a beta regression model we investigated the predictors of willingness to be vaccinated for the participants who had not yet received the vaccination offer. As shown in Table 4, the generation of pro- and against-vaccination reasons strongly influences the willingness to be vaccinated. The predicted probability from the combination of pro- and against-vaccination reasons is shown in Fig. 2 (and Table S4): respondents who did not report any reasons had an average predicted probability above 60%, while the presence of at least one reason against vaccination decreased the willingness to be vaccinated, in particular in the case of strong multiple against vaccination reasons. On the other hand, the presence of at least one pro-vaccination reason strongly increased the probability. In the end, the presence of both strong multiple pro and against vaccination reasons resulted in a high probability of getting the vaccine. Regression models adjusted by propensity score weighting allowed us to comment the influence of potential confounders: males reported an increased willingness to be vaccinated (vs. females; OR: 1.26, 95%CI: 1.11–1.42), and so did those with a high educational level (vs. low; OR: 1.22, 95%CI: 1.04–1.44) while the opposite was true among no key workers (vs. key workers; OR: 0.85, 95%CI: 0.72–0.99).

**Table 4:** Odds ratios (ORs) estimated by the weighted beta regression model for the willingness to be vaccinated with respect to the category of reference^a^. Statistically significant results are represented in bold

*Predictors*	*OR*	*95% CI*	*P-value*
Pro-vaccination reasons [Low; 1/2/3 Motiv.]	1.50	0.52; 4.35	0.456
Pro-vaccination reasons [High; 1 Motiv.]	2.30	1.85; 2.86	** < 0.001**
Pro-vaccination reasons [High; 2/3 Motiv.]	2.36	1.82; 3.06	** < 0.001**
Against-vaccination reasons [Low; 1/2/3 Motiv.]	0.17	0.10; 0.30	** < 0.001**
Against-vaccination reasons [High; 1 Motiv.]	0.20	0.14; 0.27	** < 0.001**
Against-vaccination reasons [High; 2/3 Motiv.]	0.12	0.08; 0.18	** < 0.001**
Age-class [≤ 25]	0.90	0.72; 1.12	0.334
Age-class [26-45]	0.91	0.79; 1.05	0.183
Age-class [66–84]	1.03	0.83; 1.28	0.777
Gender [Male]	1.26	1.11; 1.42	** < 0.001**
Educational level [Low]	0.95	0.83; 1.08	0.416
Educational level [High]	1.22	1.04; 1.44	**0.014**
Occupational status [Health worker]	0.85	0.64; 1.13	0.277
Occupational status [Not at work]	1.03	0.88; 1.21	0.710
Occupational status [Not health worker—Entrepreneur]	0.94	0.76; 1.16	0.557
Occupational status [Not health worker—Other]	0.92	0.73; 1.17	0.510
Familial status [Married/In a relation]	1.01	0.86; 1.18	0.904
Familial status [Divorced/Separated/Other]	0.94	0.74; 1.19	0.612
Key worker [No]	0.85	0.72; 0.99	**0.043**
Key worker [I don’t know]	0.87	0.70; 1.09	0.221
Pro-vacc. reasons [Low; 1/2/3 Motiv.]^a^Against-vacc. reasons [Low; 1/2/3 Motiv.]	2.84	0.74; 10.9	0.129
Pro-vacc. reasons [High; 1 Motiv.]^a^Against-vacc. reasons [Low; 1/2/3 Motiv.]	5.33	2.88; 9.84	** < 0.001**
Pro-vacc. reasons [High; 2/3 Motiv.]^a^Against-vacc. reasons [Low; 1/2/3 Motiv.]	5.78	3.09; 10.8	** < 0.001**
Pro-vacc. reasons [Low; 1/2/3 Motiv.]^a^Against-vacc. reasons [High; 1 Motiv.]	0.65	0.20; 2.14	0.478
Pro-vacc. reasons [High; 1 Motiv.]^a^Against-vacc. reasons [High; 1 Motiv.]	1.93	1.29; 2.89	**0.001**
Pro-vacc. reasons [High; 2/3 Motiv.]^a^Against-vacc. reasons [High; 1 Motiv.]	3.44	2.12; 5.59	** < 0.001**
Pro-vacc. reasons [Low; 1/2/3 Motiv.]^a^Against-vacc. reasons [High; 2/3 Motiv.]	1.27	0.38; 4.24	0.692
Pro-vacc. reasons [High; 1 Motiv.]^a^Against-vacc. reasons [High; 2/3 Motiv.]	1.27	0.73; 2.21	0.396
Pro-vacc. reasons [High; 2/3 Motiv.]^a^Against-vacc. reasons [High; 2/3 Motiv.]	2.76	1.67; 4.56	** < 0.001**
Observations	1289		
R^2^	0.309		

^a^reference category: Age-class [46-65], Gender [Female], Occupational status [Not health worker—Employer], Educational level [Medium], Familial status [Single/In a relation], Key worker [Yes], Pro-vaccination reasons [Missing; Invalid], Against-vaccination reasons [Missing; Invalid]

**Fig. 3:**
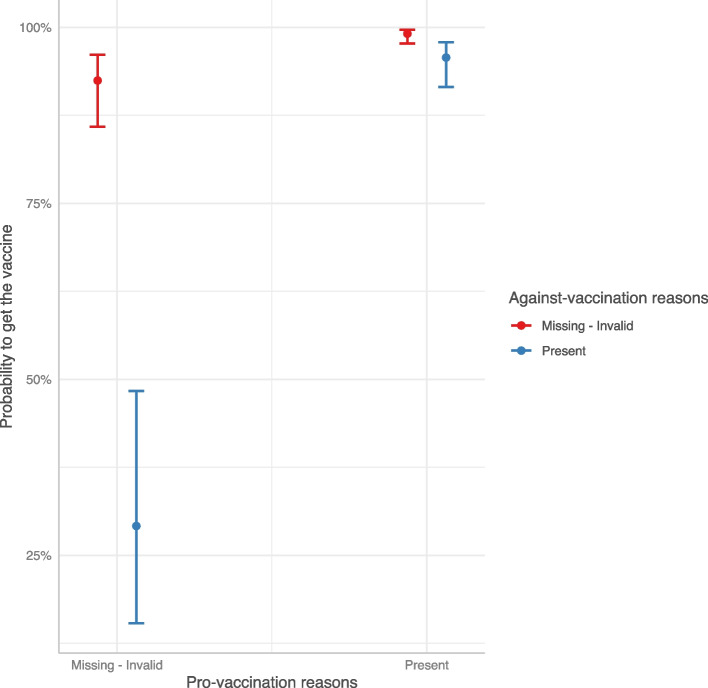
Predicted COVID-19 vaccine acceptance/booking probability by interaction between pro- and against-vaccination reasons

Finally, with a logistic model we investigated the predictors of vaccine acceptance\booking. As shown in Table 5, people who accepted or booked the COVID-19 vaccine were more likely to show pro-vaccination reasons and less likely to show against-vaccination reasons. Interestingly, when both kinds of reasons were provided, the probability of getting/booking the vaccine remained nevertheless very high (Fig. 3). Compared to the age class [46-65], younger age classes reported a strong reduction in the probability to have accepted/booked the vaccine. Male participants (OR: 1.53, 95%CI: 1.10–2.12) and those with a high educational level (OR: 2.65, 95%CI: 1.60–4.54) showed an increased probability of vaccine acceptance/booking when compared to females and participants with medium educational level, respectively. Being a health worker had a strong and positive influence on the probability of getting/booking the vaccine with respect to those employed as no health workers (OR: 6.61, 95%CI: 2.10–30.9).

**Table 5:** Odds ratios (ORs) estimated by the logistic model for the vaccine acceptance\booking with respect to the category of reference^a^

Predictors	OR	95% CI	*P*-value
Pro-vaccination reasons [Present]	8.96	4.44 – 20.4	**< 0.001**
Against-vaccination reasons [Present]	0.03	0.02 – 0.06	**< 0.001**
Age-class [≤ 25]	0.07	0.04 – 0.13	**< 0.001**
Age-class [26-45]	0.14	0.09 – 0.22	**< 0.001**
Age-class [66–84]	1.81	0.76 – 5.00	0.210
Gender [Male]	1.53	1.10 – 2.12	**0.011**
Educational level [Low]	0.57	0.40 – 0.81	**0.002**
Educational level [High]	2.65	1.60 – 4.54	**< 0.001**
Occupational status [Health worker]	6.61	2.10 – 30.9	**0.004**
Occupational status [Not at work]	1.15	0.76 – 1.75	0.510
Occupational status [Not health worker—Entrepreneur]	1.00	0.61 – 1.64	0.990
Occupational status [Not health worker—Other]	0.51	0.27 – 0.95	**0.033**
Familial status [Married/In a relation]	1.22	0.83 – 1.79	0.304
Familial status [Divorced/Separate/Other]	0.68	0.37 – 1.25	0.210
Key worker [No]	0.90	0.60 – 1.35	0.617
Key worker [I don’t know]	0.58	0.33 – 1.01	0.052
Pro-vacc. reasons [Present]^a^Against-vacc. reasons [Present]	6.03	2.13 – 16.4	** < 0.001**
Observations	392		
R^2^ Tjur	0.350		

^a^reference category: Age-class [46-65], Gender [Female], Occupational status [Not health worker—Employer], Educational level [Medium], Familial status [Single/In a relation], Key worker [Yes], Pro-vaccination reasons [Missing; Invalid], Against-vaccination reasons [Missing; Invalid]

### Classification tree analysis

**Fig. 4:**
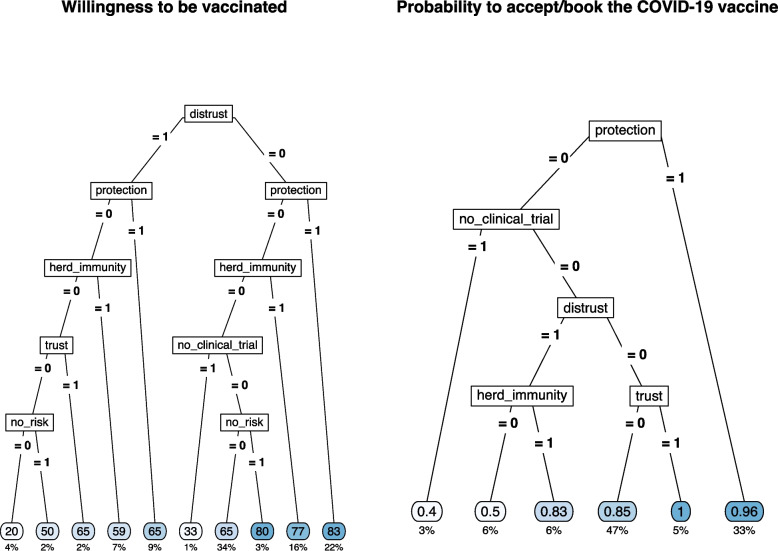
Regression tree for the willingness to be vaccinated (left) and for COVID-19 vaccine acceptance/booking (right) by selected type of pro- and against-vaccination reasons

Two regression tree models were estimated separately on the willingness to be vaccinated for those who had not yet received the vaccine offer and on the booking/acceptance of the vaccination in case of vaccine offer. Results are shown in Fig. 4. Considering the willingness to be vaccinated, the presence of distrust in the vaccination was the most discriminant variable; this latter in conjunction with reasons related to protection, herd immunity, and the absence of no clinical trials guided the willingness to be vaccinated. In particular, the combination of the absence of reasons related to distrust and the presence of protection reasons showed the highest values on the intention to get vaccinated (average value = 83 points, 22% of the sample). On the other side, the presence of at least one reason related to distrust without any positive reasons concerning protection, herd immunity, and trust predicted the lowest willingness to be vaccinated (average value = 29 points, 6% of the sample).

The sense of protection given by the vaccine or the trust in the vaccination was the main reason for vaccination acceptance/booking (average probability = 0.96 and 1.00, 33% and 5% of the sample, respectively). The combination of the absence of protective reasons and the presence of doubts about the lack of clinical studies results in the lowest likelihood of accepting/booking the vaccination (average probability = 0.40, 3% of the sample). The presence of distrust and the belief in herd immunity were the other discriminant reasons with intermediate results in terms of the probability to accept/book the vaccination.

The frequency of each category of pro- and against-vaccination reasons by COVID-19 vaccine status is shown in Table S5.

## Discussion

In the present study we aimed to investigate the reasons behind the decision to get (or not) vaccinated against COVID-19 by asking participants to report up to three reasons in favor and three reasons against the COVID-19 vaccination and to indicate the weight these reasons had in their decision. Although some researchers discourage categorization, the sparsity of the responses related to the number of reasons and their weight implies a semi-quantitative solution since a simple variable multiplication between rating and frequency (recoding to zero in case of zero reasons) is not feasible. In this case, this approach was not satisfactory as such coding would not allow differences underlying identical scores to emerge. For example, only 1 strong motivation (rating 5) would be coded in the same way as three motivations with weights 1, 2, and 2. Instead, we decided to categorize the combination of frequency-weight reasons as categorical variables (missing/invalid, low 1/2/3 reasons, high 1 reason, high 2/3 reasons) in which rating and number of reasons are combined into a single variable. This categorization allows us not only to study the weight that different categories have on the decision to get vaccinated but also to overcome the risk of imputing a specific value for missing responses.

As shown in Fig. 1, analyses were run in two steps. The first step aimed to assess how emotional competences and risk perception impacted the generation of reasons pro- and against-vaccination (Hypotheses 1A and 1B), whereas the second step investigated how different reasons affected the intention to get vaccinated (Hypotheses 2 and 3). The results support the hypotheses that emotional competences and risk perception play a significant role. Regarding emotional competence as measured by the S-PEC, the results show that high intra-personal emotional competence positively influences the production of stronger and more numerous pro-vaccination and against-vaccination reasons (confirming Hypothesis 1A). This result suggests that greater awareness of one's emotions and of what one is feeling promotes higher fluency in the production of reasons about the vaccination. Research has shown that people can be ambivalent about vaccines and hold both positive and negative reasons [2, 44]. It is reasonable to assume that, compared to people with low intra-personal emotional competences, those with high intra-personal emotional competences are more likely to have higher awareness of these contrasting attitudes and to embrace them without suppressing one of the two stances. Furthermore, the results showed that only high inter-personal emotional competences influence the generation of multiple strong reasons in favor of vaccination, and this appears to be related to the perception of vaccines as a public good and a tool to protect others. As for risk perception, a moderate to high perception of risk associated with COVID-19 influences the generation of strong pro-vaccination reasons (confirming Hypothesis 1B). These results are in line with the literature showing that a high perception of risk associated with COVID-19 positively influences the decision to get vaccinated [30, 31, 45–47]. In particular, perceiving a medium/high risk leads to generating a high number of reasons strongly in favor of vaccination, while reducing the number and weight of the reasons against the vaccine. The main premise of the psychological literature examining the relationship between risk perception and affect is that one’s behaviors are affected by rapid and intuitive evaluations, either positive or negative, people make while assessing things happening around them [48, 49]. Thus, an event is evaluated not only on the basis of objective information, but also on the basis of the experienced feelings. Emotional competence, which is clearly related to affect, also modulates how we perceive and process the emotional component underlying our judgments [36].

The results also show that, compared with younger people, those over 45 more frequently produce reasons in favor of vaccines while those over 65 produce fewer reasons against vaccination. These results are in line with the fact that younger people are at lower risk of severe consequences than older people [50], but can also be explained by considering that age was particularly salient during the period of the data collection, as the vaccination campaign was phased out by age groups, starting from the elderly. As for gender, women produced less strong pro-vaccine and weak-against vaccine reasons than men. These results are congruent with the general findings in the literature on vaccine hesitancy showing that females are more hesitant than males [5, 51, 52]. Furthermore, medium and high educational levels favored the production of both pro- and against-vaccination reasons, whereas not being in a relationship or being divorced/separated increased the generation of a strong reason against vaccination. Consistent with previous work [53], we confirmed that non-health professionals participants or non-key workers categories showed a lower intention to get vaccinated and a higher likelihood of having refused the vaccine compared to health care and key workers.

Once the role of demographics aspects and individual differences on the generation of reasons pro and/or against vaccination had been established, we ran two additional models to assess the role that those reasons have on the decision to accept the vaccination (see Fig. 1). More specifically, we tested the hypothesis that a higher number of pro- (vs. against-) vaccination reasons, connoted by a higher weight, corresponded to a stronger (vs. weaker) acceptance of vaccination (Hypothesis 2). Since data collection took place between March and April 2021, when the vaccination campaign had already started in Italy, we developed two different regression models, with the first investigating the willingness to be vaccinated in participants who were not yet offered the vaccine and the second investigating the likelihood of accepting/booking or refusing the vaccine in those who already received the offer. In particular, thanks to the propensity score weighting technique, we managed to reduce the estimates bias, especially for those factors (age, occupational status, and educational level) that influenced the vaccine offer the most [54]. The results of the two models are very similar, as the intention to get vaccinated and the likelihood of having accepted/booked the vaccine are predicted by the same factors. Specifically, the production of strong positive reasons increases either the intention to get vaccinated or having accepted/booked the vaccination. In contrast, generating strong negative reasons reduces vaccination intention and predicts the refusal of the vaccination. Hypothesis 2 is thus confirmed.

Results on the interactions between reasons, pro- and against-vaccination, and vaccination intention or vaccination choice are particularly worthy of attention. The third hypothesis was derived from the literature on prospect theory [25, 26], suggesting that at equal intensity subjective losses are more important in determining a decision than subjective gains. We therefore expected that negative reasons would count more than positive reasons in deciding whether to get vaccinated or to accept the vaccine. However, in contrast to our hypothesis, the results showed that just the generation of a single positive reason with a strong weight was enough to shift behavior and attitude in favor of the vaccination, regardless of the number and weight of negative reasons. In other words, vaccine refusal is predicted by the absence of any positive strong reasons, while when people generate both positive and negative reasons, the positive ones seem to yield a particularly important role when having a strong weight. According to prospect theory, people evaluate their goals depending on the reference point they focus on. During the pandemic, the vaccination offered an opportunity to be safer, reduced the risk of infection, and more generally appeared as the best way to re-open and get back to life as it was before COVID-19. After a year of pandemic characterized by periods of lockdown and some re-opening attempts, people were likely feeling in a state of loss (e.g., the lost freedom to go out and meet with friends and family, the lost freedom of traveling) and were looking forward to whatever chance available to recover and return to their previous lifestyle and habits. Just as those who gamble are willing to do anything to make up for a loss, so probably those who were not entirely certain about the vaccine were more willing to take risks to recover the loss in quality of life. It follows that the pandemic emergency made people forgo some of their doubts about the vaccine when, at the same time, they had reasons to get their shot. In addition, several studies [19, 55, 56] have highlighted the relationship between anticipated regret and vaccination, showing that anticipated regret is associated with an increased likelihood of adhering, or having one's children adhere, to vaccine offerings. Trusting that the vaccine would work, focusing less on its potential side effects, made sense for people who were looking forward to recovering what was perceived (and was indeed) a loss of quality of life and freedom, because they desired to be back doing the things had ever enjoyed doing (e.g., going to restaurants, movies, etc.). This finding is also interesting from a communicative perspective: providing positive reasons that resonate well with people and have therefore a strong weight for them could offset their doubts, yielding to a greater acceptance of COVID-19 vaccination.

Therefore, it is crucial to consider what kind of reasons drive the decision toward or against vaccination. Allowing participants to openly report their reasons pro- or against- vaccination can facilitate a freer exploration of the concerns and reservations of the most hesitant individuals [24], thus providing valuable insights for shaping future vaccine-related communications. In fact, thanks to the regression tree on vaccination intention, it emerges that positive attitudes toward vaccines are strongly determined by "Protection" and "Community Protection" reasons. The fact that the sense of individual and collective protection is among the principal determinants of the decision with respect to COVID-19 vaccines suggests that in general vaccination is seen as a means of avoiding nefarious clinical consequences. The effect of the sense of communal protection as the reason favoring vaccination and of other-oriented S-PEC in determining the generation of multiple pro-vaccine motivations confirms previous results suggesting that people often are more willing to get vaccinated primarily to protect their loved ones [57–59], especially when they have a good understanding of how community immunity works [60, 61]. However, it is worth mentioning that, at the time the study was conducted (March–April 2021), there was still uncertainty about whether COVID-19 vaccines could provide sterilizing immunity (i.e., could prevent the transmission of the infection) in addition to protecting the individual. To foster people's willingness to get vaccinated, it is crucial from a public health perspective that people understand that even when vaccines do not yield sterilizing immunity, vaccination can still increase protection of others by reducing the circulation of the virus.

The reasons that influenced the willingness to be vaccinated or the vaccination acceptance/booking were generally in line with the existing literature, although they differed depending on whether respondents had already been offered a vaccine or not: among those who did not received a vaccination offer, the main reasons promoting vaccination acceptance were protection against COVID-19 for oneself, one's family, friends, and community [23], while among the main reasons that reduced vaccination adherence for those who got the vaccine offer we found the lack of clinical trials [62, 63], as well as the distrust of institutions and science [22]. This latter emerged as the most reported negative reason by those who have refused the vaccine and those who have not yet received the vaccine offer. Thus, effective communication aimed at defusing the perception of risk regarding vaccines themselves should focus on enhancing trust in the scientific process and experimental rigor. Indeed, these reasons were deemed as very important not only by those who refused the vaccination, but also by those who had not yet been offered the vaccine, and even by those who held mixed feelings but eventually chose to get vaccinated. While it is unlikely that individuals firmly against vaccination will be persuaded by simple interventions [64], we should keep in mind that vaccine hesitancy is a dynamic process. As such, reducing hesitancy or enhancing ambivalence, for example through motivational interviewing (e.g., [65, 66]), could potentially lead to small shifts towards greater vaccine acceptance.

Our findings are also in line with the results of other international studies that have used a qualitative approach to examine reasons for and against vaccinations. For example, Hamilton and colleagues [67] employed a qualitative content analysis to extract the main motivations for and concerns about COVID-19 vaccination from medical records obtained by 102 consults in Australia. The study was conducted in June 2021, and revealed that most consults were driven by doubts about the vaccine available and recommended at that time (i.e., ChAdOx1-S, also known as Vaxzevria), followed by need for further information regarding vaccines and vaccination, also considering specific comorbidities. Notwithstanding the peculiarity of the Australian context in which a very low number of COVID-19 infections was observed, the analysis performed by Hamilton et al. [67] revealed a set of themes that largely overlaps with the reasons identified in our study. Indeed, among the reason to get vaccinated, 5 themes emerged: a) Protection, b) Occupational or facility responsibility or requirement, c) Trust in primary healthcare physician, d) Autonomy, and e) Civic duty, likewise, concerns about vaccination were mainly in terms of: a) Perceived vaccine risks, b) Perceived vaccine performance, c) Uncertainty, d) Autonomy, and e) Fairness in access. An aspect worth noting is that after the consultation, 81% of participants received the vaccination, 19% did not. Consistent results were observed in another study by Purvis and colleagues [68] conducted in the USA, which focused specifically on “hesitant adopters”, i.e. those who accepted vaccination but showed some level of hesitancy. To note that in this study the focus was on factors influencing the decision to get the COVID-19 vaccine, not on reasons against it. The authors interviewed 49 participants as a follow up of a larger study (*N* = 2022) conducted from mid-September 2021 through mid-October 2021, to explore factors that influenced their decision-making process about COVID-19 vaccination [68]. Two main themes emerged, each with four subthemes: 1) sociocultural context (political, cultural, health professionals, employment, and media environment) and 2) individual and group influences (attitudes and beliefs related to vaccines, family and social networks, free to return to normal, and COVID-19 outcomes).

As for the Italian context, to the best of our knowledge, only one study (i.e., [69]) attempted to provide a qualitative examination of the concept associated with vaccination in general, through open-ended and closed questions. Notably, this study was conducted a year later than our own study (April–May 2022) and was administered to a non-representative sample of Italians. The authors used a combination of closed and open-ended questions to assess concepts associated with vaccination in general. Consistent with our findings, Boragno et al. reported that participants who had been vaccinated against COVID-19 (92% of the sample) frequently mentioned concepts related to protection and salvation, whereas those who were not vaccinated frequently mentioned mistrust and ambivalence as concepts associated with vaccination [69].

This study has some limitations. First, COVID-19 perceived risk score was obtained only with respect to the disease and a similar score should be of interest for the COVID-19 vaccine. Second, data were collected during a vaccine offer limited to a well-defined slice of the population and the investigation on the vaccine acceptance/booking has, as a consequence, a limited sample size. Finally, the lack of a longitudinal perspective does not allow us to evaluate how strong the association is between the willingness to get vaccinated, vaccine acceptance and potential changes in risk perception. Thus, we cannot generalize our results beyond the period of data collection and to other countries or health systems. Since the dynamics have now changed, results may not apply to the decision to get a booster shot or not or an annual shot, however it might be interesting to study what motivations are most relevant now. Likewise, it remains to be established whether our results are generalisable to other populations.

Future studies could consider how the interaction between perceived risk associated with the disease and perceived risk associated with the vaccine influences the choice to get the shot. Furthermore, it would be important to explore how we can harness the reasons that most hold back vaccination in a specific communication strategy for the most hesitant people. Moreover, at the time of data collection, the vaccination campaign was still at an early stage, and only a small portion of the population had already received their shot. Therefore, we believe that it might be of particular interest to know more in detail, with a larger sample, what are the reasons that to date, almost 2 years after the release of the vaccine, still make some people reject the vaccine. Only by knowing these reasons will it be possible to develop appropriate vaccination campaigns.

## Conclusion

In conclusion, our work examined pro- and against-vaccination reasons and how these, and their interaction, influence the decision to get vaccinated or not. Specifically, high emotional competence and risk perception influence the generation of pro- and against-vaccination reasons and that the presence of a strong pro-vaccination reason shifts intention toward vaccination. We also highlighted the category of reasons that influence intention to vaccinate. That said, given that the discussion about the next doses is still open and that in any case the next pandemic is a matter of when and not if [70], it is of paramount importance to know the best way to counteract vaccine hesitancy, fostering more effective communication strategies.

### Supplementary Information

**Additional file 1: Appendix 1.** Scoring for pro- and against-vaccination reasons. **Appendix 2.** Structure of the questionnaire. **Table S1.** Selection criteria. **Table S2.** Number of items, internal consistency (Cronbach’s α), name of the items and their estimated loadings, total deviance explained by the loadings and proportion of variance explained by EFA for COVID-19 perceived risk. **Table S3.** Odds ratios (ORs) estimated by the logistic model for the propensity score weighting for the COVID-19 vaccine offer. **Table S4**. Predicted willingness to get vaccinated by combination of pro- and against-vaccination reasons by category of reference. **Table S5.** Frequency of reported categories of pro- and against-vaccination reasons overall, and by COVID-19 vaccine status. **Figure S1.** Distribution of the propensity scores by vaccine offer.

## Data Availability

Raw data are available on https://osf.io/dpn2q/?view_only=af05427467634411b471af7a8475ffab.
