# Supplementary material for: To vaccinate or not to vaccinate? The interplay between pro- and against- vaccination reasons
Source: BMC Public Health. 2023 Nov 9;23:2207. doi: 10.1186/s12889-023-17112-6 (PMC10634164; doi:10.1186/s12889-023-17112-6)
Supplement: Supplementary file 1 — Additional file 1: Appendix 1. Scoring for pro- and against-vaccination reasons. Appendix 2. Structure of the questionnaire. Table S1. Selection criteria. Table S2. Number of items, internal consistency (Cronbach’s α), name of the items and their estimated loadings, total deviance explained by the loadings and proportion of variance explained by EFA for COVID-19 perceived risk. Table S3. Odds ratios (ORs) estimated by the logistic model for the propensity score weighting for the COVID-19 vaccine offer. Table S4. Predicted willingness to get vaccinated by combination of pro- and against-vaccination reasons by category of reference. Table S5. Frequency of reported categories of pro- and against-vaccination reasons overall, and by COVID-19 vaccine status. Figure S1. Distribution of the propensity scores by vaccine offer. [file 12889_2023_17112_MOESM1_ESM.docx]

**To vaccinate or not to vaccinate? The interplay between pro- and against- vaccination reasons**

**Supplementary material**

**Contents:**

| 1. Appendix 1: Scoring for pro- and against-vaccination reasons. | Page 2 |
| --- | --- |
| 1. Appendix 2: Structure of the questionnaire. | Page 3 |
| 1. Table S1. | Page 4 |
| 1. Table S2. | Page 4 |
| 1. Table S3. | Page 5 |
| 1. Table S4. | Page 5 |
| 1. Table S5. | Page 6 |
| 1. Figure S1. | Page 7 |

1. **Appendix 1. Scoring for pro- and against-vaccination reasons.**

| **Categories in favor of vaccines** | | **Categories against vaccine** | |
| --- | --- | --- | --- |
| **Category** | **Example** | **Category** | **Example** |
| **Trust** | - “Sicuro” (R_psj967STrkezoqZ)  [“Safe”]  - “Mi fido dei ricercatori e della medicina”  (R_1esZbqmESAuhP4U)  ["I trust researchers and medicine."] | **Distrust** | - “non sempre è efficace” (R_2YzyrdBEJSIQz77)  [“is not always effective”]  - “non mi fido di questa situazione” (R_1oFRhPD7Q4jlBku)  [“I don't trust this situation”] |
| **Community immunity** | - “per arrivare il prima possibile all’immunita’’ (R_2AZlvlcZOmESjt2)  [“To get to immunity as soon as possible”]  - “far finire la pandemia” (R_1dit6OF6oCKlhia)  [“end the pandemic”] | **Lack of risk perception** | - “preferisco alimentare le mie difese immunitarie con alimentazione e integratori” (R_3Rsj0MDVqljS1Do)  ["I prefer to feed my immune system with nutrition and supplements."]  - “Non sono attualmente in una situazione di rischio” (R_3kFmbBLZH7jVnsd)  [“I am not currently in a risk situation”] |
| **COVID-19-related risk** | - “protezione da complicanze in caso di contagio” (R_3sblvLdlZ58L28y)  ["protection from complications in case of infection"]  - “è meno rischioso vaccinarsi che contagiarsi” (R_AbnaOdlkgBRJaKF)  ["it's less risky to vaccinate than to get infected."] | **Vaccine health risks** | - “allergia a qualche ingrediente” (R_2162T3bSKrsNtQG)  "allergy to some ingredient"  - “effetti collaterali” (R_3M9YEkYkx4KRXUq)  ["side effects"] |
| **Protection**  **& Prevention** | - “Utile per gli anziani”  (R_2dNmZGGkqASXRtY)  ["Useful for seniors."]  - “Utile per i più fragili” (R_0qd1GqelaHt2Wjf)  ["Useful for the most fragile."] | **Sociopolitical concerns** | - “non ha avuto sperimentazione" (R_3M9YEkYkx4KRXUq)  ["had no experimentation"]  - “Ti possono impiantare i microchip” (R_1ghdFcB3aRpYroc)  ["They can implant you with microchips."] |
| **Positive emotional aspects** | - “fa stare più tranquilli” (R_2YzyrdBEJSIQz77)  ["makes people feel more comfortable."]  - “toglie un poco di ansia quando si deve uscire” (R_C9yBn9WrUvbrU8F)  ["It takes away a little anxiety when you have to go out."] | **Negative emotional aspects** | - “ho paura” (R_2xyjJlDQCoefhZV)  ["I am scared"]  - “Non mi sento sicura” (R_1ly4POohazkOrbU)  ["I don't feel safe."] |

1. **Appendix 2. Structure of the questionnaire.**

- Please report your gender: Male, Female, I do not identify with any of the above, I prefer not to answer.
- Age (in number)
- Familiar status: single/in a relation, married/cohabitant, divorced/separated/other
- Educational level: low= middle school or lower, medium = high school, high = degree or higher
- Occupational status: public employee, private employee, freelancer, entrepreneur/entrepreneur, retired person, unemployed, student, other (specify).*
- Key worker status: yes, no, I don’t know
- Health worker: yes, no
- Past COVID-19 contagion: no, yes asymptomatic, yes low symptoms, yes severe symptoms
- Have you already had one of the available vaccines against COVID-19? *Yes (at least the first dose); Not yet, but I have made the reservation; No, I have not yet been contacted; No, I have declined*
- **(IF No, I have not yet been contacted) Willingness to vaccinate**: Imagine that today you had to decide whether to vaccinate against COVID-19. Would you vaccinate yourself? Answer by moving the slider from 0 = *Not at all likely*, to 100 = *Extremely likely*.
- **Reason pro vaccination:** Write at least one reason pro the vaccine. Then rate how important that reason is in your decision to vaccinate or not to vaccinate against COVID-19; 1 = Not at all, 5 = Extremely.
- **Reason against vaccination:** Write at least one reason against the vaccine. Then rate how important that reason is in your decision to vaccinate or not to vaccinate against COVID-19; 1 = Not at all, 5 = Extremely.
- **Risk perception - item 1:** On a scale of 0 to 100, how scared do you feel about the Coronavirus?
- **Risk perception - item 2:** On a scale of 0 to 100, how serious do you think the illness caused by Coronavirus is?
- **Risk perception - item 3:** On a scale of 0 to 100, how likely do you think it is that you will get sick (or, if you have already gotten sick, get sick again) from the Coronavirus?
- **Risk perception - item 4:** On a scale of 0 to 100, how concerned are you about possible mutations (or variants) of the Coronavirus?
- 20-item Profile of Emotional Competence scale (PEC; Mikolajczak et al., 2013)

******the information about the job, in combination with information on work in the health sector, was recoded as: health worker (any worker at work in health sector), not at work (retired, unemployed, student), not health worker-employer (private/public employee not at work in health sector), not health worker-entrepreneur (entrepreneur/entrepreneur not at work in health sector), not health worker-other (freelance/other not at work in health sector).*

1. **Table S1. Selection criteria.**

| **Selection criteria n (%)** | **N = 1,833** |
| --- | --- |
| Survey completed | 1,756 (96%) |
| Presence of missing values | 112 (6.1%) |
| Response not valid | 16 (0.9%) |
| Included | 1,681 (92%) |

1. **Table S2. Number of items, internal consistency (Cronbach’s α), name of the items and their estimated loadings, total deviance explained by the loadings and proportion of variance explained by EFA for COVID-19 perceived risk.**

| **N. of Items** | 4 |
| --- | --- |
| ***Cronbach's* α** | 0.852 |
| **Item value range** | 1-100 |
| **Item 1** | Scared |
| **Item 2** | Severity |
| **Item 3** | Contagious |
| **Item 4** | Mutation |
| **Loadings item 1** | 0.856 |
| **Loadings item 2** | 0.790 |
| **Loadings item 3** | 0.582 |
| **Loadings item 4** | 0.854 |
| **SS loadings** | 2.43 |
| **% Var. explained** | 61 |

1. **Table S3. Odds ratios (ORs) estimated by the logistic model for the propensity score weighting for the COVID-19 vaccine offer.**

| **Predictors** | **OR** | **95% CI** | ***P*-value** | |
| --- | --- | --- | --- | --- |
| Age-class [26-45] | 0.85 | 0.53; 1.40 | 0.527 | |
| Age-class [46-65] | 1.18 | 0.73; 1.95 | 0.506 | |
| Age-class [66-84] | 3.83 | 2.21; 6.72 | **<0.001** | |
| Gender [Female] | 0.84 | 0.64; 1.08 | 0.173 | |
| Occupational status [Not at work] | 0.11 | 0.06; 0.20 | **<0.001** | |
| Occupational status [Not health worker - Employer] | 0.15 | 0.09; 0.26 | **<0.001** | |
| Occupational status [Not health worker - Entrepreneur] | 0.09 | 0.04; 0.17 | **<0.001** | |
| Occupational status [Not health worker - Other] | 0.11 | 0.05; 0.22 | **<0.001** | |
| Educational level [Middle] | 1.30 | 0.98; 1.73 | 0.069 | |
| Educational level [High] | 1.80 | 1.28; 2.52 | **0.001** | |
| Key worker [No] | 0.62 | 0.45; 0.86 | **0.004** | |
| Key worker [I don’t know] | 0.63 | 0.39; 0.99 | **0.049** | |
| Past COVID-19 contagion [Yes, asymptomatic] | 1.21 | 0.54; 2.62 | 0.630 | |
| Past COVID-19 contagion [Yes, low symptoms] | 0.58 | 0.32; 0.99 | 0.053 | |
| Past COVID-19 contagion [Yes, severe symptoms] | 1.11 | 0.15; 5.52 | 0.903 | |
| Familial status [Married/Cohabitant] | 1.14 | 0.82; 1.60 | 0.451 | |
| Familial status [Divorced/Separate/Other] | 1.53 | 0.94; 2.47 | 0.082 | |
| Observations | 1681 | | |  |
| R^2^ Tjur | 0.134 | | |  |

**reference category: age-class [18-25], Gender [Male], Occupational status [Health worker], Educational level [Low], Key worker [Yes], Past COVID-19 contagion [No, I don’t known], Familial status [Single/In a relation].*

1. **Table S4. Predicted willingness to get vaccinated by combination of pro- and against-vaccination reasons by category of reference***

|  | **Pro-vaccination reasons** | | | |
| --- | --- | --- | --- | --- |
| **Against-vaccination reasons** | Missing - Invalid | Low; 1/2/3 reasons | High; 1 reason | High; 2/3 reasons |
| Missing - Invalid | 64.3 | 73.0 | 80.6 | 81.0 |
| Low; 1/2/3 reasons | 23.7 | 57.0 | 79.2 | 80.9 |
| High; 1 reason | 26.2 | 25.7 | 61.2 | 74.3 |
| High; 2/3 reasons | 18.0 | 29.6 | 39.1 | 58.9 |

**reference category: age-class [46-65], Gender [Female], Occupational status [Not health worker - Employer], Educational level [Medium], Familial status [Single/In a relation], Key worker [No], Pro-vaccination reasons [Missing; Invalid], Against-vaccination reasons [Missing; Invalid].*

1. **Table S5. Frequency of reported categories of pro- and against-vaccination reasons overall, and by COVID-19 vaccine status.**

The belief in herd immunity and the sense of protection offered by the vaccine were the most frequent categories of pro-vaccination reasons, and they appeared less frequently reported by those refusing the vaccine with respect to the other categories of respondents, respectively: *p*=0.012 for herd immunity and *p*<0.001 for the sense of protection. The presence of health issues was the most reported reason against vaccination which appears more frequent among those who have not yet received the vaccination offer (34%), whereas those who refused the vaccination exhibited the lowest frequency of that reason (17%). A statistically significant difference in the frequency of no clinical trial and distrust was observed in COVID-19 vaccine status, respectively *p*=0.015 and *p*<0.001.

| **Pro-vaccination reasons** | **Overall**,  N = 1,681*^1^* | **COVID-19 vaccine status** | | | | ***P*-value***^2^* |
| --- | --- | --- | --- | --- | --- | --- |
|  |  | **Booked**,  N = 159*^1^* | **Done**,  N = 179*^1^* | **Not yet**,  N = 1,289*^1^* | **Refused**,  N = 54*^1^* |  |
| Trust | 153 (9.1%) | 14 (8.8%) | 22 (12%) | 115 (8.9%) | 2 (3.7%) | 0.264 |
| Herd immunity | 539 (32%) | 43 (27%) | 54 (30%) | 434 (34%) | 8 (15%) | **0.012** |
| No risk | 169 (10%) | 12 (7.5%) | 22 (12%) | 131 (10%) | 4 (7.4%) | 0.501 |
| Protection | 535 (32%) | 50 (31%) | 75 (42%) | 405 (31%) | 5 (9.3%) | **<0.001** |
| Positive emotions | 36 (2.1%) | 4 (2.5%) | 1 (0.6%) | 30 (2.3%) | 1 (1.9%) | 0.444 |
| **Against-vaccination reasons** |  |  |  |  |  |  |
| Distrust | 380 (23%) | 21 (13%) | 29 (16%) | 312 (24%) | 18 (33%) | **<0.001** |
| Invulnerable | 15 (0.9%) | 0 (0%) | 0 (0%) | 14 (1.1%) | 1 (1.9%) | 0.217 |
| Health issues | 557 (33%) | 48 (30%) | 56 (31%) | 444 (34%) | 9 (17%) | **0.028** |
| No clinical trial | 139 (8.3%) | 7 (4.4%) | 8 (4.5%) | 116 (9.0%) | 8 (15%) | **0.010** |
| Negative emotions | 54 (3.2%) | 9 (5.7%) | 1 (0.6%) | 42 (3.3%) | 2 (3.7%) | **0.041** |
| *^1^*Median (IQR) or Frequency (%)  *^2^* Fisher’s Exact Test for Count Data with simulated p-value (based on 2000 replicates) | | | | | | |

1. **Figure S1. Distribution of the propensity scores by vaccine offer.**

**
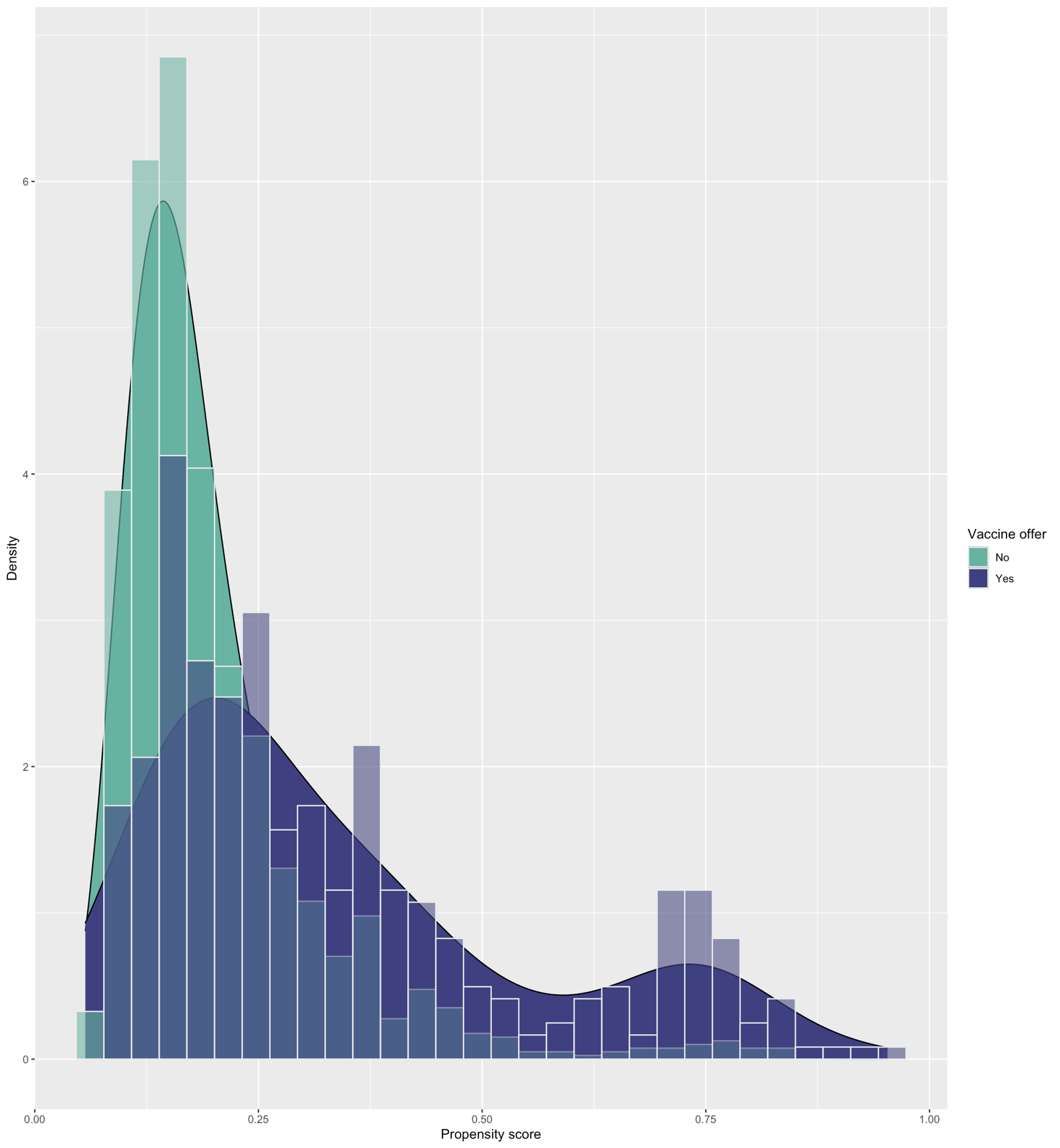
**
